# Wear Characteristics of WC-Co Cutting Tools Obtained by the U-FAST Method During Particleboard Milling

**DOI:** 10.3390/ma18163907

**Published:** 2025-08-21

**Authors:** Joanna Wachowicz, Zbigniew Bałaga, Piotr Podziewski

**Affiliations:** 1 Institute of Wood Sciences and Furniture, Department of Mechanical Processing of Wood, Warsaw University of Life Sciences, Nowoursynowska Street, 166, 02-787 Warsaw, Poland; piotr_podziewski@sggw.edu.pl; 2 Faculty of Production Engineering and Materials Technology, Czestochowa University of Technology, Armii Krajowej Street, 19, 42-201 Czestochowa, Poland; zbigniew.balaga@pcz.pl

**Keywords:** cemented carbides, WC-Co, sintering, cutting tools, particleboard machining

## Abstract

This article presents the wear characteristics of the working surface of WC-Co (Tungsten Carbide–Cobalt) tungsten carbide tools obtained using the innovative U-FAST (Upgraded Field-Assisted Sintering Technology) method for particleboard machining. Three groups of tools with a similar chemical composition but differing WC (Tungsten Carbide) grain sizes were tested. Milling tests were carried out on a CNC (Computer Numerical Control) machine tool with the following cutting parameters: spindle rotation at 15,000 rpm, a feed rate of 0.25 mm per tooth, and a feed rate of 3.75. The experimental results show that tools with submicron WC grit sizes of 0.4 µm and 0.8 µm have the longest tool life. Wear of the cutting edges occurred through the removal of the cobalt bond between the tungsten carbide grains, leading to fracture and mechanical removal of the grains from the cutting edge surface. The similarities in the relative wear characteristics of blades with submicron tungsten carbide grain sizes suggest that micro-abrasion and bond phase extrusion may be the main wear mechanisms under the experimental conditions. Nanometric WC grain size significantly influences tool wear through chipping and cracking.

## 1. Introduction

The furniture industry is a significant component of the Polish economy. It is an established fact that particleboard is one of the most widely utilized materials within the furniture industry. The manufacturing process of particleboard entails the compression of wood shavings, which are impregnated with resins, under precise pressure and temperature conditions. The chips used for the production of boards are obtained by mechanical cutting from healthy fine wood, waste wood, or wood that is not suitable for processing into boards. Particleboard boasts a number of advantages, including dimensional stability, good strength characteristics, and resistance to temperature and moisture fluctuations. It is unfortunate that, in addition to wood in particle or fibre form, binders, fillers and thinners, particleboard and fibreboard also contain silica [1,2,3,4,5].

In the domain of material machining, particularly in the context of milling processes, it is imperative to select the optimal cutting tools to ensure superior machining quality, process efficiency, and tool life. The utilisation of WC-Co composites in the machining of challenging materials is a common practice, owing to the composites’ elevated hardness, wear resistance, and dimensional stability. Carbide tools are most commonly used for machining wood-based materials. Cutting tools are frequently exposed to elevated temperatures and high levels of intensity during operation. The application of transverse pressure has been demonstrated to enhance the vulnerability of such tools to fracture at temperatures that exceed ambient levels. The durability of carbide tools is contingent upon the material properties that are exhibited at elevated temperatures [6].

Cemented carbides are constituted of a fine-grained metal carbide in a metal binder. The majority of cemented carbides are of the tungsten carbide variety, with a cobalt WC-Co binder. Carbides are conventionally manufactured through a process involving the cold pressing of powder mixtures, followed by sintering in the liquid phase within induction or resistance-heated furnaces. The temperature of these furnaces is typically in the range of 1300 to 1600 °C, with the precise temperature depending on the composition of the carbide mixture. Research has demonstrated that the sintering mechanism is contingent on the dimensions of WC particles and the quantity of binder that is dispersed across the particle boundaries of carbides [7]. In conventional carbide technologies, VC, Cr3 C2 inhibitors are incorporated into the sintering process with a view to constraining the growth of WC grains, which have been observed to expand when exposed to elevated temperatures. In the work of [8], WC-Co composites were prepared using the conventional sintering method with the incorporation of inhibitors. In the case of superfine WC-Co formulations containing both vanadium carbide and chromium carbide, the admixture of a grain growth inhibitor was equally effective. The results indicate that the presence of VC has a tendency to reduce the solubility of W in the Co alloy, thus inhibiting WC dissolution and growth [9,10,11]. It has been demonstrated that the hardness and strength of carbides improve as the WC grain size decreases to nanometre levels. Consequently, ultrafine-grained carbide exhibits superior mechanical properties in comparison with conventional alloys. However, during the preparation of ultrafine-grained carbide, crystalline grains exhibit a heightened propensity for growth during the sintering process. This phenomenon can be attributed to the lower surface energy exhibited by nano-WC particles in comparison with their ordinary WC counterparts. Consequently, the conventional sintering method often proves ineffective in the production of ultrafine WC grains. At present, two principal methodologies are employed for the purpose of inhibiting the growth of WC grains. These methodologies are the addition of grain growth inhibitors and the selection of a fast sintering process [12,13].

Standard sintering technology is subject to certain limitations in terms of producing composites with ultrafine and nanometric WC grain sizes for cutting-edge applications. These limitations are a result of the process parameters, which include high temperatures and extended durations. The presence of inhibitors has been demonstrated to have a dual effect on grain growth: whilst they limit the process, they concomitantly increase grain hardness and reduce resistance to brittle fracture [14,15,16,17]. Consequently, FAST (field-assisted sintering technique) sintering methods are employed, utilising pulses of electric current for the purpose of heating. This technique facilitates the synthesis of powder materials at comparatively low temperatures and in a relatively brief timeframe [18,19,20]. The process under discussion is innovative in nature, and is characterised by a reduction in energy consumption and an acceleration in the consolidation process. In addition, it exhibits a low energy consumption ratio, ranging from one-fifth to one-third of that observed in conventional sintering techniques. The technology is characterised by high efficiency, which is attributable to the direct heating of the compressed powder materials using high-powered pulsed direct current. The Joule heat released during the process results in uniform heating, surface cleaning and activation of individual particles of the powder mixture. It is evident that these sintering conditions facilitate the attainment of a homogeneous and dense structure [21,22,23,24]. The U-FAST method belongs to the FAST (field-assisted sintering technology) group. Thanks to its advanced heating system based on a unique pulse shape, the U-FAST device achieves better results than other available FAST devices. Additionally, the pulse duration of less than 1 ms gives U-FAST an advantage over SPS devices. Previous tests of U-FAST materials have revealed their potential in applications where other methods cannot achieve the required material quality.

WC-Co carbides are most commonly used in the woodworking industry as blade materials, inserts for circular saws and peripheral milling machines. Notwithstanding their extensive utilisation, there remains a paucity of knowledge concerning their deterioration, particularly in the context of high-velocity machining of wood-based materials. In the process of cutting wood and wood-based products, a number of mechanisms have the potential to contribute to the deterioration of cutting tools. In the context of wear mechanisms, the most prevalent include abrasion, erosion, cracking, splintering, micro-cracking, chemical corrosion, and oxidation. Cracking instigates sudden or catastrophic failure of the cutting edge, while other wear mechanisms engender gradual or progressive wear. The chemical wear of WC-Co blades has been the subject of study in the following work [25,26,27]. Wear was found to occur as a result of preferential dissolution of the cobalt binder by chemical attack of the extracts present in the green wood. This, in turn, leads to the mechanical removal of the tungsten carbide grains.

Cutting tool wear is a pivotal parameter that exerts a significant influence on the economic viability and quality of the machining process. The Archard model can be used to quantify wear. However, it does not consider the mechanisms that accompany tool wear processes [28]. The wear process is a complex one, the resultant parameters of which are dependent on a number of factors. These include the properties of the workpiece material, the geometry of the tool, the cutting parameters, and the technology employed in the manufacture of the tool. It is imperative to comprehend the wear mechanisms of tools composed of WC-Co composites, obtained by the U-FAST method, when milling chipboard, a material that is extensively utilized in the furniture and wood-based industries. In the present study, a particleboard is processed using preset milling parameters with WC-Co carbide tools. The WC grain sizes obtained using the innovative U-Fast sintering method vary. The objective of this study is to characterize the wear process of WC-Co cutting tools produced by the U-FAST method during chipboard milling. The conclusions of this study are focused on the optimization of the machining process and the identification of opportunities to increase the efficiency and cost-effectiveness of the technology.

## 2. Materials and Methods

### 2.1. Tool Material

The tool materials included three types of WC-Co carbides, obtained by U-FAST technology (GeniCore Sp. z o.o., Warsaw, Poland), in the works [29,30], with different WC grain sizes and similar cobalt binder content. The fundamental properties are outlined in Table 1. Subsequent to sintering, the samples exhibited a diameter of 20 mm and a thickness of 1.5 mm. Subsequently, blades measuring 12 × 12 mm were shaped through the utilization of an electric spark saw.

### 2.2. Workpiece

**Figure 1:**
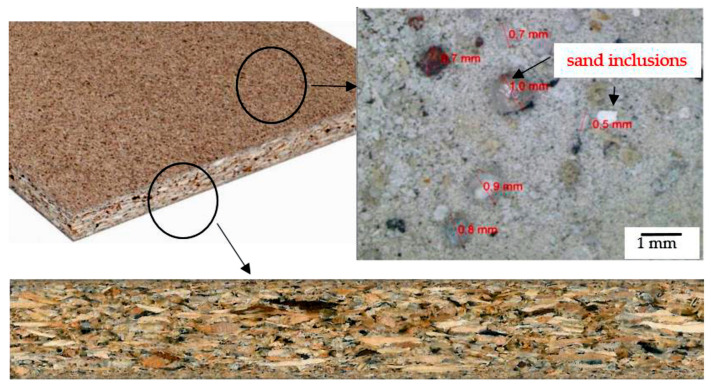
Three-layer particleboard [32].

A three-layer particleboard was used for the durability tests. Table 2 summarizes the basic properties of the machined material, and Figure 1 shows a photograph of it. The particleboard sheets to be tested measured 400 × 1000 mm.

### 2.3. Wear Measurement

**Figure 2:**
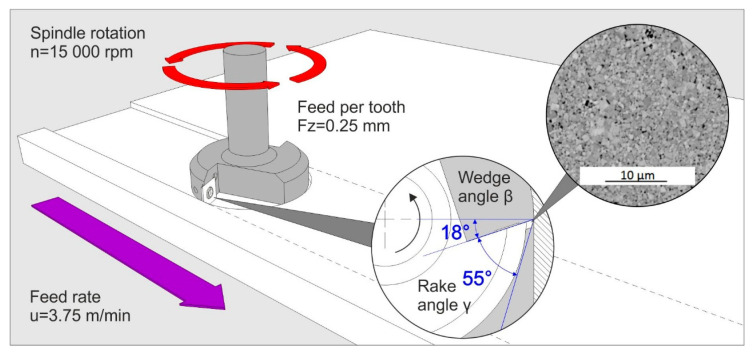
Schematic diagram of experiment.

The cutting edges of the cutting inserts were ground along all four sides, at an angle of 55°. The milling tests were conducted using a CNC machining centre, specifically the Busselatto Jet 100 (Busellato S.p.A., Thiene, Italy). The depth of cut was 4 mm. The machining parameters utilized are outlined in Table 3. The abrasion of the contact surface was measured at intervals of 1 m along the feed distance, which corresponded to the length of the particleboard. The condition of the blade was meticulously monitored utilizing a shop microscope until the dullness criterion of VBmax = 0.2 mm was attained. The milling process is schematically shown in Figure 2.

### 2.4. Cutting Edge Analysis

Following the conclusion of the milling process, the carbide blades were extracted from the tool holder, and the cutting edge was subjected to scrutiny using a JEOL 6610LV (JEOL Ltd., Tokyo, Japan) scanning microscope equipped with a LaB6 cathode and a Keyence VHX-6000 (Keyence Corporation, Osaka, Japan) digital optical microscope at a perpendicular angle to the cutting surface. The cutting edge was traced along its entire length, and photographs of the wear profile were taken. The extent of wear on each blade was determined by measuring the area under the wear profile.

## 3. Results and Discussion

**Figure 3:**
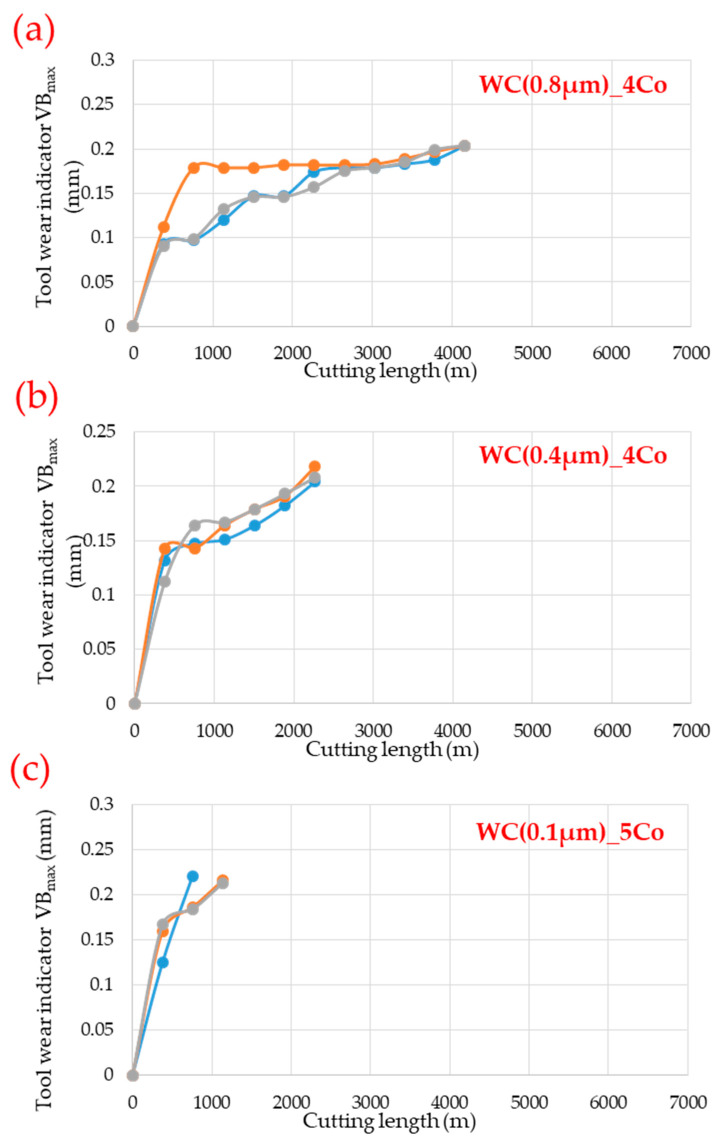
Tool wear curves of: (**a**) WC(0.8 µm)_4Co; (**b**) WC(0.4 µm)_4Co; (**c**) WC(0.1 µm)_5Co composites [33].

Figure 3 shows sample wear curves for the tested blades, which were developed as part of the work [33]. The tools were withdrawn from further testing once they reached the established wear criterion of 0.2 mm. The curves obtained for the WC-Co 0.8 µm tools were similar to the classic wear progression curve (Lorentz curve), i.e., the curve showing the running-in, steady-state, and acceleration phases. Using these blades did not modify the nature of the wear curve; for example, it did not eliminate the running-in phase. In the case of blades with WC grain sizes of 0.4 µm and 0.1 µm, analysis of the curves indicates two-period wear behaviour during which the intensity of wear changes.

**Figure 4:**
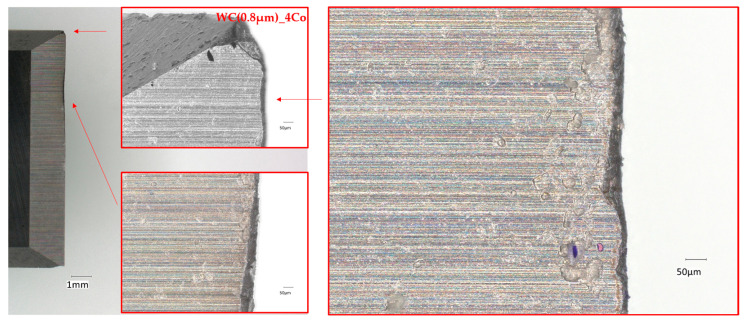
Micrographs of the WC(0.8 µm)_4Co worn edges when cutting particleboard.

**Figure 5:**
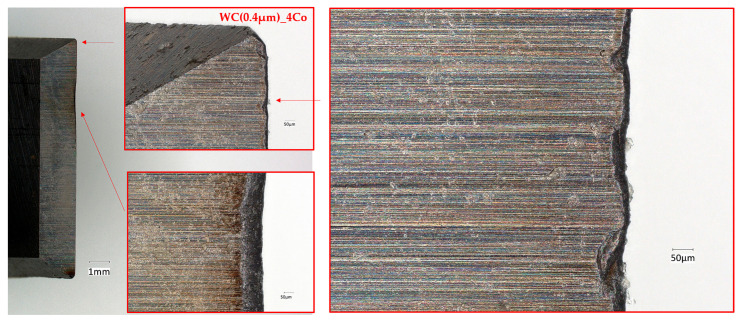
Micrographs of the WC(0.4 µm)_4Co worn edges when cutting particleboard.

Figure 4 and Figure 5 illustrate optical microscope images of a worn edge that had been produced following the milling of a chipboard with blades manufactured from carbide with submicron WC grain sizes of 0.4 and 0.8 µm, respectively. These images reveal that wear of the cutting edge occurred on the work surface as a result of friction between the work surface and the workpiece. Concurrently, they render the uniform progressive wear of the tool edge perceptible.

**Figure 6:**
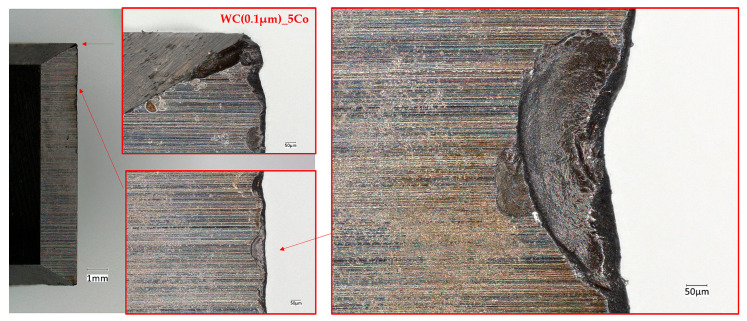
Micrographs of the WC(0.1 µm)_5Co worn edges when cutting particleboard.

Conversely, tools exhibiting a nanometric WC grain size of 0.1 µm (Figure 6) demonstrate a high prevalence of breakouts and chipping along the edge, ultimately leading to severe tool degradation. At the nanoscale, the material’s structure becomes more complex, with greater effects associated with grain boundaries appearing. This can cause an uneven distribution of internal stresses. The fine-grained structure of nanometric carbides can lead to localised stresses at grain boundaries, potentially causing cracking. Additionally, materials with nanometre-sized grains may exhibit defects such as dislocations and cracks. The presence of these defects, combined with the conditions during the milling process, can exacerbate mechanical stresses.

**Figure 7:**
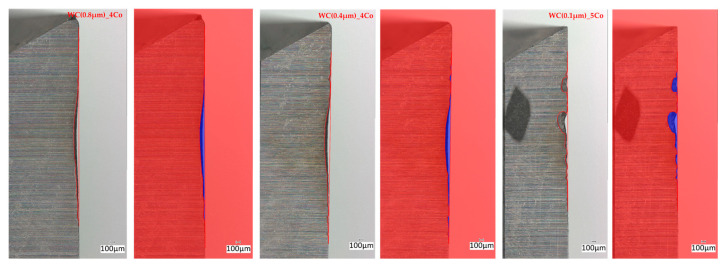
Variation of edge wear along the length of the cutting edge.

The wear rate of each variant of the tested blades, as determined by the area under the wear profile, was found to be similar across all variants (Figure 7 and Table 4). It is possible to discern variations in the configuration of such a profile. It has been demonstrated that the finer the grain, the more numerous the chipping and breakage of the cutting edge.

**Figure 8:**
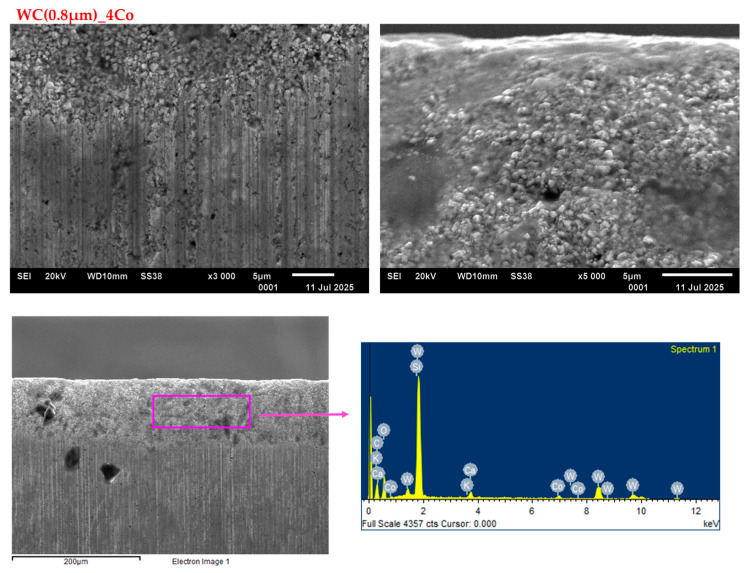
SEM images of the WC(0.8 µm)_4Co worn edges when cutting particleboard.

**Figure 9:**
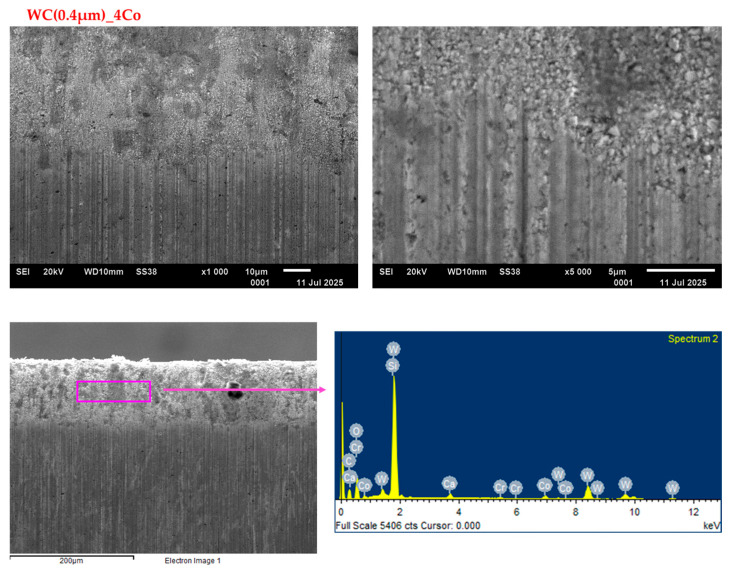
SEM images of the WC(0.4 µm)_4Co worn edges when cutting particleboard.

Figure 8 and Figure 9 illustrate SEM images of the worn edges of 0.8 and 0.4 µm blades, respectively. A thorough examination of the SEM micrographs indicated that the deterioration of the cutting edge predominantly occurred on the working surface, a consequence of the friction between the working surface and the workpiece. SEM images obtained at higher magnifications reveal the presence of tungsten carbide grains that protrude convexly from the wear surface. In addition, the images show depressions that were once occupied by carbide grains, but had since been removed. It is hypothesised that, for blades with grain sizes of 0.4 and 0.8 µm, wear occurs through the removal of the cobalt bond between the WC grains, which then leads to the removal of the WC grains themselves. The observation that the wear surfaces of submicron WC grain sizes are similar in appearance suggests that analogous wear mechanisms. Numerous scientific studies confirm that the preferential removal of tungsten carbide grains during the wear process is a complex, multifaceted phenomenon occurring through various physicochemical mechanisms. The most important of these are chemical corrosion, oxidation, extrusion, and abrasion. In practice, it is highly likely that multiple mechanisms are at work simultaneously during the wear process. These mechanisms overlap and interact, creating a complex picture of material degradation. For instance, oxidation can form protective layers or weak layers that accelerate the wear process.

**Figure 10:**
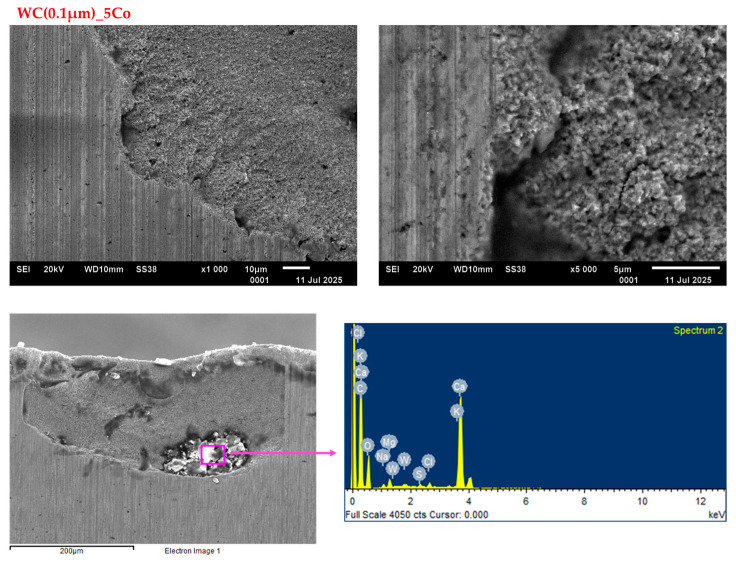
SEM images of the WC(0.1 µm)_5Co worn edges when cutting particleboard.

In the case of blades made of cemented carbides with nanometric WC grain sizes, the presence of distinct microcracks was observed, which ultimately led to catastrophic blade wear (Figure 10). A salient disadvantage of this material is its low resistance to the milling parameters employed. Durability tests were also conducted as part of another study [34]. The experiment encompassed the testing of WC-Co composites, incorporating WC grain sizes ranging from 0.7 to 1.7 µm. The reduction in grain size and bonding phase content has been demonstrated to result in a reduction in mean free path and an increase in hardness, leading to an increase in wear resistance. However, the utilisation of 0.7 µm grains diminished the resistance of this material to strong impacts and heavy cutting. The absence of impact resistance renders such materials unsuitable for utilisation under particularly exacting cutting conditions. Consequently, the authors proposed the utilisation of an enhanced cutting insert design, entailing modifications to the geometry and the refinement of the quality of the cutting edge through the employment of modified grinding techniques.

Blades with nanometre-sized WC grains can fracture or break off fragments due to mechanical action, resulting in a deterioration in machining quality. Mechanical stresses are caused by forces during the cutting process, such as pressure, impact, or vibration. These forces can weaken the blade’s structure, particularly in weak or damaged areas. To minimize this risk, it is necessary to regularly monitor blade condition, use appropriate machining parameters, and use durable, stress-resistant materials.

The EDS analysis (Figure 8, Figure 9 and Figure 10) of all the tested blade variants revealed the presence of elements such as calcium, silicon, and oxygen, in addition to tungsten, carbon, and cobalt. These elements are not part of the tool material itself, but rather constitute impurities within the chipboard, such as silicon or calcium from calcium hydroxide. Alternatively, they may be introduced during the production stage as regulators and hardeners. The presence of these elements in the analysed blade edge may therefore indicate that, in addition to the predominant wear mechanism through abrasion, there is also an adhesion phenomenon.

Cobalt (occurring in tools) is susceptible to oxidation, particularly at the high temperatures that can be reached during milling processes. Oxidation can weaken the mechanical properties of cobalt tools, reducing their wear resistance, for example. The formation of oxides acts as a defect in the material structure and contributes to premature tool wear. However, it should be noted that the presence of oxygen in EDS analysis does not always clearly indicate the presence of oxidised phases; natural oxidation of tool surfaces during storage can also cause oxygen to be detected.

## 4. Conclusions

The impact of tool wear on machining quality is a key consideration in machining processes, since the condition of the tools directly affects both the end result and production efficiency.

As worn tools lose their original cutting properties, uneven material removal can occur. This can result in surfaces with nicks, scratches, or other defects that reduce the part’s aesthetic appeal and functionality. Such surface irregularities may necessitate additional finishing processes, thereby increasing production time and costs. Tool wear also reduces machining precision, which can result in dimensional distortion of parts. When cutting tools lose their sharpness, uncontrolled material distortion can occur, affecting dimensional tolerances and the quality of the final product. More frequent tool changes are associated with higher costs for purchasing and maintaining tool inventory. Increased wear and tear can force production rates to slow down in order to maintain high-quality machining, thereby reducing overall plant productivity.

In summary, regular checks of tool condition and proper maintenance are key to maintaining high-quality machining. Early detection of wear enables tool replacement to be planned. The following conclusions can be drawn from the tests carried out:

1. It has been demonstrated that tools with submicron WC grain sizes of 0.4 and 0.8 µm exhibit the longest tool life.
2. The deterioration of the carbide cutting tools milling the chipboard was attributed to the preferential WC grain dropout, which was followed by fracture and chipping. The underlying wear mechanisms were identified as abrasive wear and adhesive wear.
3. The impact of WC grain size on tool wear is a significant consideration, with nanometric particles having a notable effect on tool durability due to their tendency to cause spalling and fracture.
4. It is important to continue developing WC-Co tools with nanometric WC grain sizes. Consider modifying the chemical composition or optimizing the cutting edge geometry to achieve improved strength and cutting performance.

## Figures and Tables

**Table 1:** Properties of the cemented tungsten carbide inserts used in this study.

Blades	Avg. WC Grain Size (µm)	Chemical Compositions		Mechanical Properties		Literature
Cemented Carbide		WC (wt.%)	Co (wt.%)	Hardness (HV30)	K_IC_ (MPam^1/2^)	
WC(0.8 µm)_4Co	0.8	96	4	2085	8.36	[29]
WC(0.4 µm)_4Co	0.4	96	4	2270	8.33	[29]
WC(0.1 µm)_5Co	0.1	95	5	2192	9.27	[30]

**Table 2:** Specifications of the cutting material [31].

Wood-Based Board	Density (kg/cm^3^)	Brinell Hardness	Bending Strength (%)	Modulus of Elasticity (MPa)	Sand Content (%)
Three-layer particleboard	649	2.6	8.7	2212	0.185

**Table 3:** Cutting parameters.

Spindle Rotation (rpm)	Feed Rate per Tooth, F_z_ (mm)	Feed Rate, u (m/min)
15,000	0.25	3.75

**Table 4:** Area under wear curves.

	Mean Area Under Wear Curve (mm^2^)
WC(0.8 µm)_4Co	0.26
WC(0.4 µm)_4Co	0.33
WC(0.1 µm)_5Co	0.31

## Data Availability

Data are contained within the article. Further inquiries can be directed to the corresponding author.

## References

[1] Darmawan W., Rahayu I., Nandika D., Marchal R. (2012). The importance of extractives and abrasives in wood materials on the wearing of cutting tools. BioResources.

[2] Porankiewicz B., Gronlund A., Lemaster R. Tool wear—Influencing factors. Proceedings of the 10th International Wood Machining Seminar.

[3] Bendikiene R., Keturakis G. (2017). The influence of technical characteristics of wood milling tools on its wear performance. J. Wood Sci..

[4] Nasir V., Cool J. (2020). A review on wood machining: Characterization, optimization, and monitoring of the sawing process. Wood Mater. Sci. Eng..

[5] Wei W., Li Y., Xue T., Tao S., Mei C., Zhou W., Wang T. (2018). The research progress of machining mechanisms in milling wood-based materials. BioResources.

[6] Tang X., Wang Z., Huang L., Wang X., Chang T., Huang P., Zhu Z. (2023). Preparation, properties and microstructure of high hardness WC-Co cemented carbide tool materials for ultra-precision machining. Int. J. Refract. Met. Hard Mater..

[7] Da Silva A.G.P., Schubert W.D., Lux B. (2001). The role of the binder phase in the WC-Co sintering. Mater. Res..

[8] Carroll D.F. (1999). Sintering and microstructural development in WC/Co-based alloys made with superfine WC powder. Int. J. Refract. Met. Hard Mater..

[9] Zhong Y., Shaw L.L. (2011). Growth mechanisms of WC in WC-5.75 wt% Co. Ceram. Int..

[10] Wang X., Fang Z.Z., Sohn H.Y. (2008). Grain growth during the early stage of sintering of nanosized WC–Co powder. Int. J. Refract. Met. Hard Mater..

[11] Poetschke J., Richter V., Gestrich T. (2014). Grain growth during sintering of tungsten carbide ceramics. Int. J. Refract. Met. Hard Mater..

[12] Mannesson K., Borgh I., Borgenstam A., Agren J. (2011). Abnormal grain growth in cemented carbides-experiments and simulations. Int. J. Refract. Met. Hard Mater..

[13] Mannesson K., Jeppsson J., Borgenstam A., Ågren J. (2011). Carbide grain growth in cemented carbides. Acta Mater..

[14] Gille G., Szesny B., Dreyer H., van den Berg K., Schmidt J., Gestrich T., Leitner G. (2002). Submicron and ultrafine grained hardmetals for microdrills and metal cutting insert. Int. J. Refract. Met. Hard Mater..

[15] Schubert W.D., Neumeister H., Kinger G., Lux B. (1998). Hardness to toughness relationship of fine-grained WC-Co hardmetals. Int. J. Refract. Met. Hard Mater..

[16] Imasato S., Tokumoto K., Kitada T., Sakaguchi S. (1995). Properties of ultra-fine grain binderless cemented carbide ‘RCCFN’. Int. J. Refract. Met. Hard Mater..

[17] Fang Z., Eason J.W. (1995). Study of nanostructured WC-Co composites. Int. J. Refract. Met. Hard Mater..

[18] Huang Z., Ren X., Liu M., Xu C., Zhang X., Guo S., Chen H. (2017). Effect of Cu on the microstructures and properties of WC-6Co cemented carbides fabricated by SPS. Int. J. Refract. Met. Hard Mater..

[19] Wang Z., Jia J., Wang B., Wang Y. (2019). Two-Step Spark Plasma Sintering Process of Ultrafine Grained WC-12Co-0.2VC Cemented Carbide. Materials.

[20] Buravleva A.A., Fedorets A.N., Vornovskikh A.A., Ognev A.V., Nepomnyushchaya V.A., Sakhnevich V.N., Lembikov A.O., Kornakova Z.E., Kapustina O.V., Tarabanova A.E. (2022). Spark Plasma Sintering of WC-Based 10wt%Co Hard Alloy: A Study of Sintering Kinetics and Solid-Phase Processes. Materials.

[21] Tokita M. (2021). Progress of Spark Plasma Sintering (SPS) Method, Systems, Ceramics Applications and Industrialization. Ceramics.

[22] Tokita M. (2006). Development of Advanced Spark Plasma Sintering (SPS) Systems and Its Industrial Applications. Ceram. Trans. Am. Ceram. Soc..

[23] Munir Z.A., Anselmi-Tamburini U., Ohyanagi M. (2006). The effect of electric field and pressure on the synthesis and consolidation of materials: A review of the spark plasma sintering method. J. Mater. Sci..

[24] Grasso S., Sakka Y., Maizza G. (2009). Electric current activated/assisted sintering (ECAS): A review of patents 1906–2008. Sci. Technol. Adv. Mater..

[25] Kirbach E., Chow S. (1976). Chemical wear of tungsten carbide cutting tools by Western redcedar. Prod. J..

[26] Bailey J.A., Bayoumi A.M., Stewart J.S. (1983). Wear of cemented tungsten carbide tools in machining oak. Wear.

[27] Bayoumi A.E., Bailey J.A., Stewart J.S. (1983). Comparison of the wear resistance of various grades of cemented carbides that may find application in wood machining. Wear.

[28] Coors T., Faqiri Y., Saure F., Pape F., Hassel T., Poll G. (2023). Wear of tailored forming steels. Adv. Eng. Mater..

[29] Wachowicz J., Kruzel R., Bałaga Z., Ostrowska A., Dembiczak T. (2023). Application of U-FAST Technology in Sintering of Submicron WC-Co Carbides. Materials.

[30] Wachowicz J., Fik J., Bałaga Z., Stradomski G. (2023). Testing for Abrasion Resistance of WC-Co Composites for Blades Used in Wood-Based Material Processing. Materials.

[31] Wachowicz J., Wilkowski J., Talarek S. (2023). Influence of cutting parameters on the tool life for WC-Co composites in the machining of wood-based materials. Wood Mater. Sci. Eng..

[32] Wachowicz J., Dembiczak T., Stradomski G., Bałaga Z., Dyner M., Wilkowski J. (2021). Properties of WCCo Composites Produced by the SPS Method Intended for Cutting Tools for Machining of Wood-Based Materials. Materials.

[33] Wachowicz J., Wilkowski J., Dembiczak T., Kruzel R. (2025). Durability of Cutting Tools Obtained by U-FAST Technology in Particleboard Machining. Materials.

[34] Sheikh-Ahmad J., Bailey J.A. On the wear of cemented carbide tools in the continuous and interrupted cutting of particleboard. Proceedings of the 14th International Wood Machining Seminar.

